# Hantaan Virus (HTNV) Human Infection on Jeju Island, South Korea: Unique Phylogeny and Epidemiology of HTNV

**DOI:** 10.1002/jmv.70305

**Published:** 2025-03-18

**Authors:** Misun Kim, Sang Taek Heo, Su Yeon Kang, EunJin Bae, Jeong Rae Yoo, Yoon‐Jae Song, Michael Wiley, Jessica D. Wiley, Huy Chau Nguyen, Andrew G. Letizia, Keun Hwa Lee

**Affiliations:** ^1^ Department of Internal Medicine Jeju National University College of Medicine Jeju South Korea; ^2^ Department of Microbiology Hanyang University College of Medicine Seoul South Korea; ^3^ Department of Life Science Gachon University Seongnam‐Si South Korea; ^4^ Department of Pathology Microbiology, and Immunology College of Medicine University of Nebraska Medical Center Omaha Nebraska USA; ^5^ PathoSeq Bio LLC Department Omaha Nebraska USA; ^6^ Naval Medical Research Unit INDO PACIFIC

To the Editor,

*Orthohantavirus hantanense* (Hantaan virus, HTNV) is an enveloped, tripartite, negative‐sense, and single‐stranded RNA virus (seven serotypes and genotypes of HTNV) that infects various species of rodents [1, 2]. Striped field mice (*Apodemus agrarius*) are natural reservoirs of HTNV with the virus identifiable in urine and feces [1, 2, 3, 4, 5]. Spread to humans occurs after contact with infected rodents, their droppings, urine, or possibly inhalation of virus particles in places containing large amounts of rodent droppings. HTNV can cause hemorrhagic fever with renal syndrome (HFRS), which can lead to kidney damage [1, 2, 3]. The clinical presentation of HFRS often includes fever, shock, hemorrhage, and acute renal failure [1, 2, 3]. Globally, approximately 100,000 cases of HFRS are reported annually, with the majority occurring in China, Russia, and South Korea [1, 2, 3, 4]. Although HTNV infection is frequently reported in China, each year in Taiwan, HTNV causes 0‐4 human cases of HFRS and not endemic country [5].

In South Korea, approximately 400 cases of HFRS caused by HTNV are reported per year, with a mean mortality rate of 1%–4%. Cases often come in two peaks with the majority of HTNV infection occurring between October and December and a second period from May and July. Approximately 18 HFRS cases were reported from 2011 to 2019 on Jeju Island, South Korea [3, 6]. However, human HTNV infection has not been confirmed on Jeju Island, which is located at the southern end of the Korean Peninsula and among Japan, Taiwan, and China (33°0′ N, 126°0′ E).

In this study, we found autochthonous cases of human HTNV on Jeju Island in 2024 with a clinical presentation consistent with HFRS. A total of 4 HFRS cases were laboratory confirmed and were treated at Jeju National University Hospital (JNUH) from April 2024 to June 2024 (Table 1). The Institutional Review Board of JNUH approved the study (IRB file no. 2024‐09‐028) and all the subjects signed informed consent forms. For the molecular diagnosis of HTNV infection, we performed nested PCR to amplify the partial large (L) segment of the viral RNA from the stored blood and confirm HTNV infection. We sequenced the nested PCR products (356 bp) via the BigDye Terminator Cycle Sequencing Kit (Perkin Elmer Applied Biosystems, Warrington, UK) [7, 8]. Phylogenetic analysis was performed with Bayesian inference in BEAST (v1.10.4) using the best‐fit model (GTR + I + G) identified by jModelTest (v2.1.10) (Supplementary Data).

**Table 1:** Basic characteristics of HFRS patients on Jeju Island, South Korea in 2024.

Patient	Age (years) /sex	Exposure area	Occupation	Exposure to rodents	Date of sampling	ANC (/μL)	Hct (%)	Platelet (x10³/μL)	AST (IU/L)	ALT (IU/L)	Cr (mg/dL)	LDH (IU/L)	PT (sec)	PT (INR)	aPPT (sec)	Proteinuria on dipstick test	Outcome
JP1‐2024	50/M	Jocheon‐eup,	Engineer	No	May 24	12 712	39.8	41	49	32	6.81	427	12.6	1.15	40	4+	Recovered
		Hallim‐eup,															
		Odeung‐dong															
JP2‐2024	56/M	Aewol‐eup	Farmer	No	April 24	26 838	61	34	185	50	4.68	1472	13.8	1.27	43	4+	Recovered
JP3‐2024	60/M	Ora‐dong	Unemployed	No	May 24	10 458	46.1	28	38	17	1.94	463	12.6	1.15	38	3+	Recovered
JP4‐2024	44/M	Sangye‐dong	Lawyer	No	June 24	4720	38.6	65	68	57	2.14	548	11.1	1	35.7	4+	Recovered

*Note:* The normal ranges of laboratory findings were as follows: ANC (/μL), Hct (39‐52%), platelet (150–450 × 10³/μL), AST (8–38 IU/L), ALT (4–44 IU/L), Cr (0.60–1.10 mg/dL), LDH (106–211 IU/L), PT (9.9–13.5 s), PT (INR) (0.88–1.20 INR), PT INR (0.88–1.20 INR), aPPT (20.0–36.0 s), and proteinuria [negative–trace (±)]. All the patients lived on Jeju Island and had no history of travel to the HTNV endemic region.

Abbreviations: ALT, alanine aminotransferase; ANC, absolute neutrophil count; aPTT, activated partial thromboplastin time; AST, aspartate aminotransferase; Cr, creatinine; Hct, hematocrit; INR, international normalized ratio; LDH, lactate dehydrogenase; PT, prothrombin time.

Autochthonous human HTNV infections on Jeju Island in 2024 may have occurred during a period similar to the previous period (minor epidemic periods, May and July) reported for the Korean Peninsula (Table 1) [3, 4]. All the patients lived on Jeju Island; none reported a history of travel to the HTNV endemic region for HTNV on the Korean Peninsula nor to other endemic countries, such as China or Russia; but all did report that they had visited wooded, hilly, or mountainous areas before the onset of symptoms (Table 1). Our phylogenetic analysis of these samples revealed that the viral sequences were the same as or similar to previously reported partial L segment sequences (99.7%‐100% nucleotide identity) from field mice (*Apodemus chejuensis*) collected from Jeju Island between 2018 and 2020 and we suggest that *A. chejuensis* could be the source for the human HTNV infections on Jeju Island (Figures 1 and S1a) [6, 8, 9].

**Figure 1:**
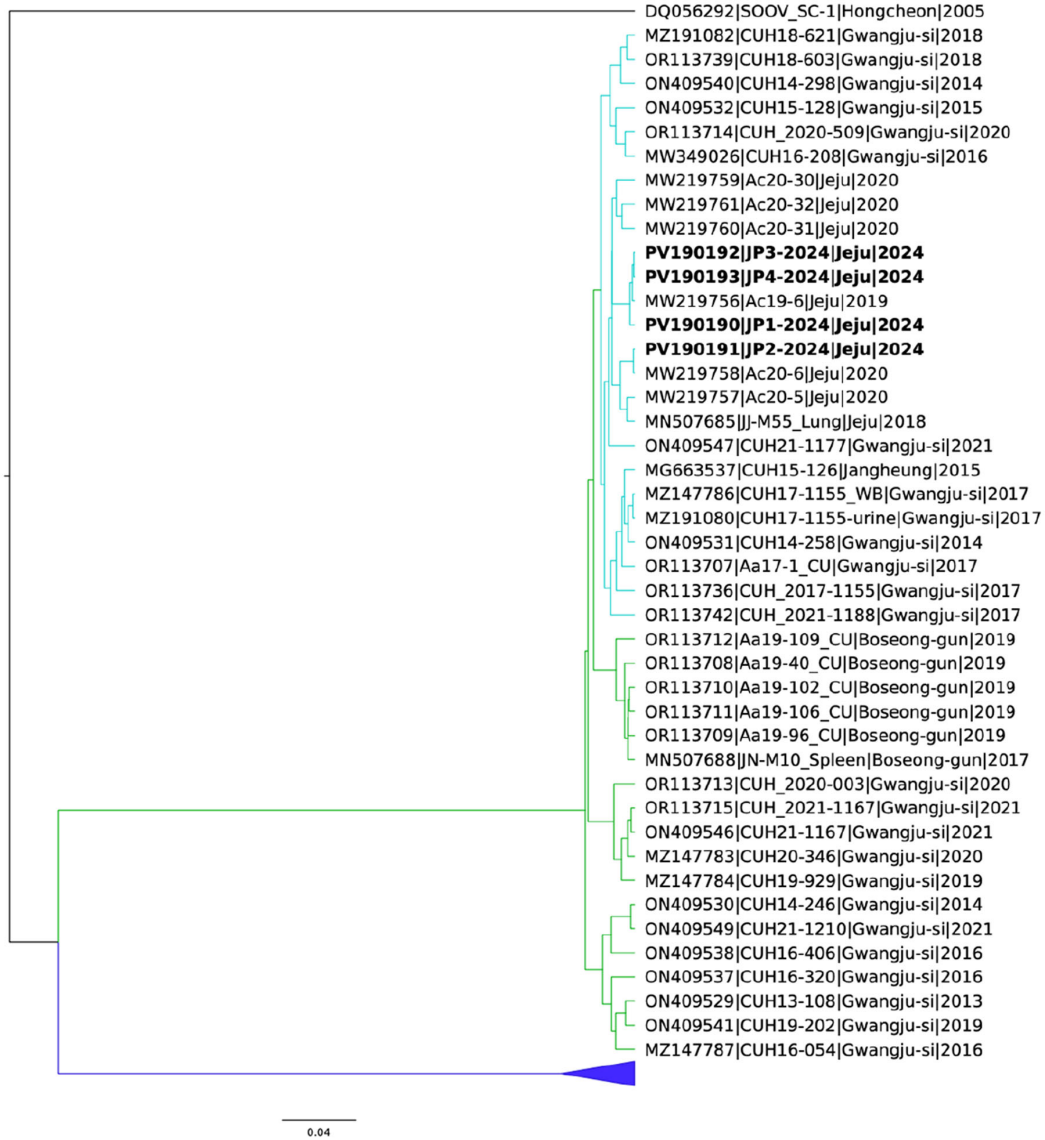
Phylogenetic analysis of South Korea HTNV L segment. The phylogenetic tree was constructed on the basis of full and partial L segment sequences (356 bp) of South Korea HTNV obtained from NCBI in addition to the four samples from this study which were identified from stored blood samples collected in 2024 from patients on Jeju Island, South Korea (bold) (GenBank accession numbers PV190190 to PV190193, respectively). Phylogenetic analysis was performed using the Bayesian inference method in BEAST (v1.10.4) based on the full HTNV L segments trimmed to the coding region (28–6504 nt) as well as other partial HTNV L segments (*n* = 164). The scale bar indicates the number of nucleotide substitutions per site. The collapsed clade (indicated by a large triangle) includes a number of sequences (Northern South Korea) that formed a separate cluster and were collapsed for readability. Soochong virus (DQ056292) was included as root sequence. Highlighting: Northern South Korea genomes, blue; Southern South Korea genomes, green; Jeju‐specific lineage, South Korea, cyan.

In conclusion, we confirmed autochthonous cases of human HTNV infection on Jeju Island in 2024 and that the genomes differed from those previously reported from the Korean Peninsula, China, and Russia and suggest that *A. chejuensis* is likely an important source of human HTNV infection on Jeju Island (Figures 1 and S1a,b). Therefore, further epidemiological, clinical, and laboratory research is needed to better understand the transmission dynamics of HTNV on Jeju Island. Additionally, human HTNV infection should be considered when acute renal failure develops in patients with fever and thrombocytopenia on Jeju Island, South Korea. Our study has several limitations. This was a nonrandomized, retrospective study involving convenience samples of a relatively small number of inpatients seen in one university hospital, and the phylogenetic analysis included only those samples collected in 2024.

## Author Contributions

Keun Hwa Lee acquired the funding. Keun Hwa Lee, Su Yeon Kang, Misun Kim, EunJin Bae, Sang Taek Heo, Michael Wiley, and Jessica D. Wiley performed the experiments and analyzed the data. Misun Kim, Sang Taek Heo, and Jeong Rae Yoo collected and provided the clinical samples. Keun Hwa Lee, Misun Kim, Sang Taek Heo, Andrew G. Letizia, Michael Wiley, Jessica D. Wiley, and Huy Chau Nguyen interpreted the data. Keun Hwa Lee, Andrew G. Letizia, Sang Taek Heo, Su Yeon Kang, and Misun Kim wrote the manuscript. Andrew G. Letizia, Michael Wiley, Jessica D. Wiley, Yoon‐Jae Song, and Huy Chau Nguyen critically reviewed the manuscript. All the authors have read and agreed to the published version of the manuscript.

## Ethics Statement

This study was reviewed and approved by the Local Research Ethics Committee of the Jeju National University Hospital (IRB file no. 2024‐09‐028). Informed consent was obtained from all patients following the principles of the Helsinki Declaration.

## Conflicts of Interest

The authors declare no conflicts of interest. The views expressed in this article reflect the results of research conducted by the author and do not necessarily reflect the official policy or position of the Department of the Navy, Department of Defense, nor the United States Government. CAPT Andrew Letizia, MC, USN and LT Huy Nguyen, MC, USN are military service members of the United States Government. This study was prepared as part of their official duties. Title 17 U.S.C. 105 provides that “copyright protection under this title is not available for any work of the United States Government.” Title 17 U.S.C. 101 defines a US Government work as work prepared by a military service member or employee of the US Government as part of that person's official duties.

## Supporting information

Supporting information.

Supporting information.

## Data Availability

The data used to support the results of this study are available from the corresponding author (Keun Hwa Lee) upon reasonable request.
