# Supplementary material for: Hantaan Virus (HTNV) Human Infection on Jeju Island, South Korea: Unique Phylogeny and Epidemiology of HTNV
Source: J Med Virol. 2025 Mar 18;97(3):e70305. doi: 10.1002/jmv.70305 (PMC11917189; doi:10.1002/jmv.70305)
Supplement: Supplementary file 1 — Supporting information. [file JMV-97-e70305-s002.pptx]

## Slide 1
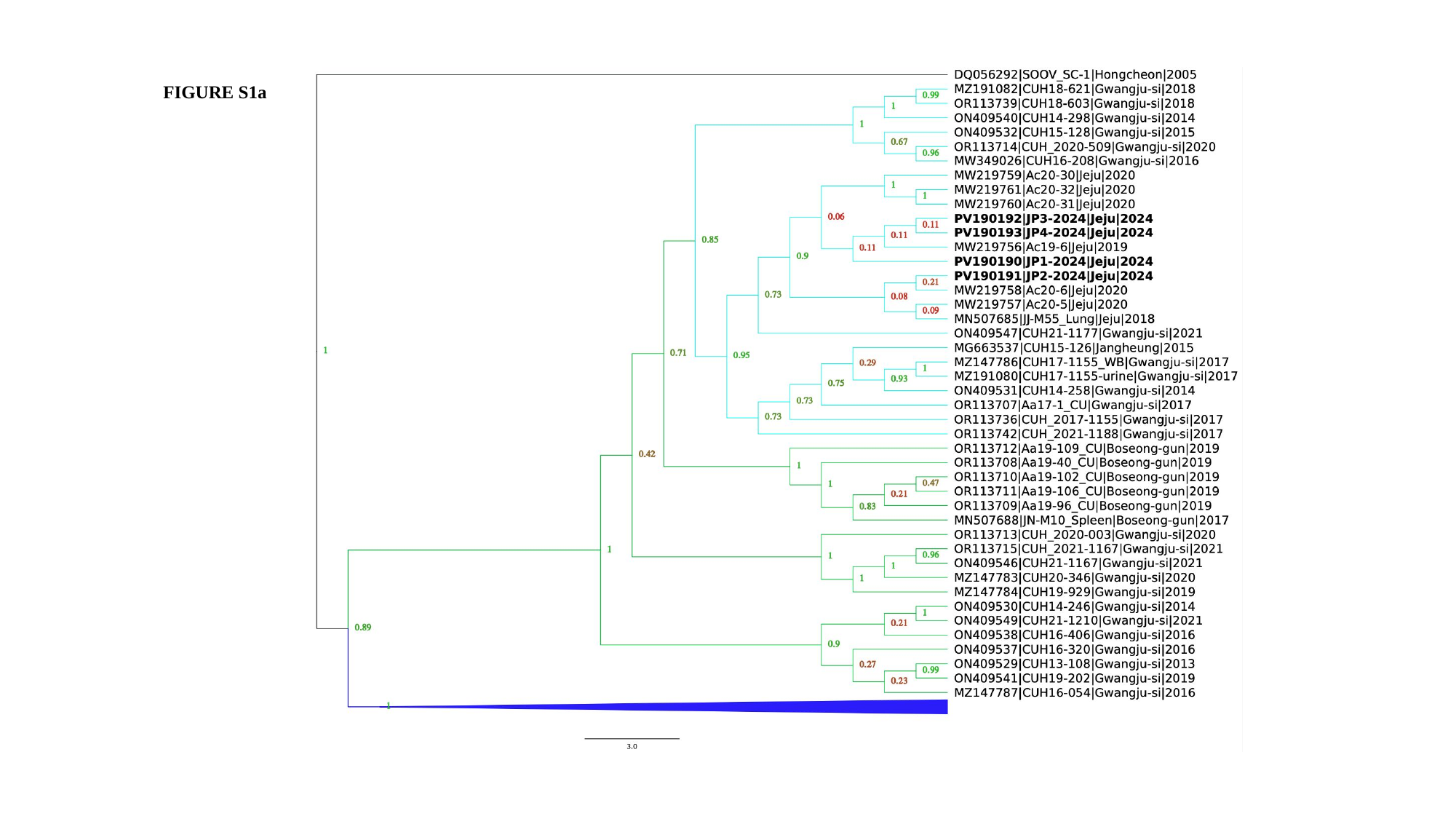

FIGURE S1a

## Slide 2
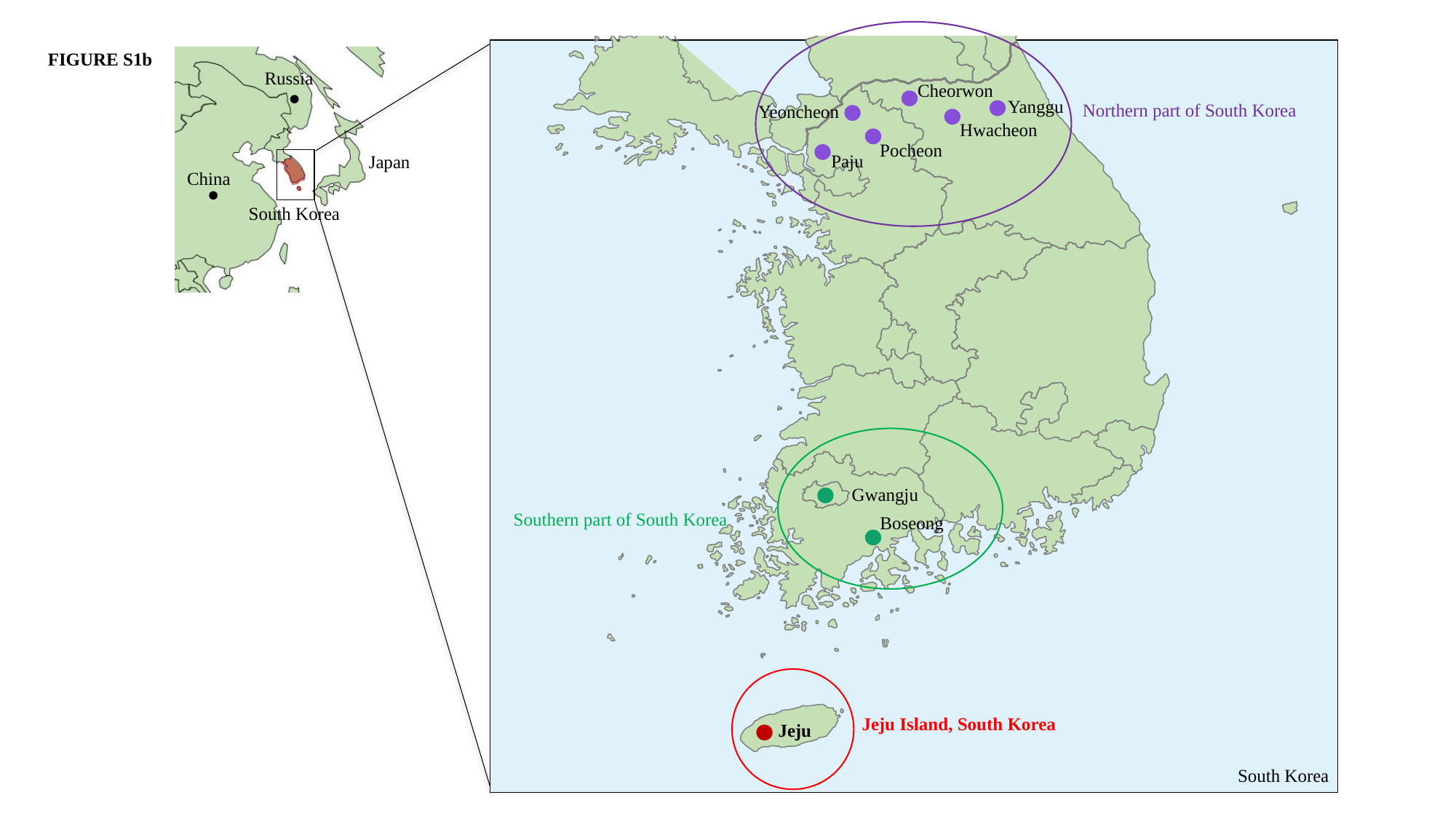

Cheorwon
Yanggu
Yeoncheon
Hwacheon
Pocheon
Paju
Gwangju
Boseong
Jeju
FIGURE S1b
Russia
Northern part of South Korea
Japan
China
South Korea
Southern part of South Korea
Jeju Island, South Korea
South Korea
