# Supplementary material for: Hantaan Virus (HTNV) Human Infection on Jeju Island, South Korea: Unique Phylogeny and Epidemiology of HTNV
Source: J Med Virol. 2025 Mar 18;97(3):e70305. doi: 10.1002/jmv.70305 (PMC11917189; doi:10.1002/jmv.70305)
Supplement: Supplementary file 2 — Supporting information. [file JMV-97-e70305-s001.docx]

**Supplementary Data** Phylogenetic analysis of South Korea HTNV L segment. Full and partial L-segment genomes of South Korea HTNV were obtained from National Center for Biotechnology Information (NCBI), along with four new partial genomes from this study. After trimming full genomes to the coding region (28-6504 nt) and filtering identical sequences, the dataset (164 unique genomes) was aligned using MAFFT (v7.490) in Geneious (v2025.0.3). Phylogenetic analysis was performed with Bayesian inference in BEAST (v1.10.4) using the best-fit model (GTR+I+G) identified by jModelTest (v2.1.10). The analysis employed an Markov Chain Monte Carlo (MCMC) chain length of 15 million states, sampling every 1000 states. Adequate sampling (ESS > 200) was confirmed, and a maximum clade credibility (MCC) tree was generated in TreeAnnotator (v1.10.4) with a 10% burn-in. The tree was visualized in FigTree (v1.4.4) with posterior probabilities overlaid and the cladogram feature applied for branch and leaf support.

**FIGURE S1a** Posterior Probabilities Cladogram Transformation of Phylogenetic Analysis of South Korea HTNV L segment (Northern South Korea collapsed). Figure 1 tree with the posterior probabilities for the topology generated using the MCMC algorithm and transformed with FigTree’s “cladogram” feature to clearly display the probabilities.

**FIGURE S1b** HTNV geographical location in South Korea, China, and Russia. The sequence metadata of the viruses were obtained from GenBank. The purple circles indicate HTNV in the northern part of South Korea, the green circles indicate HTNV in the southern part of South Korea, and the red circles indicate HTNV on Jeju Island, South Korea.
